# Improving DNA barcoding library of armored scale insects (Hemiptera: Diaspididae) in China

**DOI:** 10.1371/journal.pone.0301499

**Published:** 2024-05-30

**Authors:** Minmin Niu, Yubo Liu, Linjia Xue, Bo Cai, Qing Zhao, Jiufeng Wei

**Affiliations:** 1 College of Plant Protection, Shanxi Agricultural University, Taigu, China; 2 Hainan Province Engineering Research Center for Quarantine, Prevention and Control of Exotic Pests, Haikou, China; National Cheng Kung University, TAIWAN

## Abstract

DNA barcoding is used to identify cryptic species, survey environmental samples, and estimate phyletic and genetic diversity. Armored scale insects are phytophagous insects and are the most species-rich taxa in the Coccoidea superfamily. This study developed a DNA barcode library for armored scale insect species collected from southern China during 2021–2022. We sequenced a total of 239 specimens, recognized as 50 morphological species, representing two subfamilies and 21 genera. Sequencing analysis revealed that the average G + C content of the cytochrome oxidase subunit I (*COI*) gene sequence was very low (~18.06%) and that the average interspecific divergence was 10.07% while intraspecific divergence was 3.20%. The intraspecific divergence value was inflated by the high intraspecific divergence in ten taxa, which may indicate novel species overlooked by current taxonomic treatments. All the Automated Barcode Gap Discovery, Assemble Species by Automatic Partitioning, Taxon DNA analysis and Bayesian Poisson Tree Process methods yielded largely consistent results, indicating a robust and credible species delimitation. Based on these results, an intergeneric distance threshold of ≤ 5% was deemed appropriate for the differentiation of armored scale insect species in China. This study establishes a comprehensive barcode library for the identification of armored scale insects, future research, and application.

## Introduction

DNA barcoding is a method of species identification, using short standardized DNA fragments, first proposed by Herbert et al. in 2003 [1, 2]. It has since been used in several fields of biology, including taxonomy [3–5], ecology [6, 7], conservation biology [8], and evolution [9]. It can be used to elucidate cryptic species [10], survey environmental samples [11], and estimate phyletic and genetic diversities [12, 13]. Additionally, DNA barcoding can be used for species identification, when information on the morphology and taxonomy of the species is limited [14], such as for cryptic species and immature or mutilated specimens.

Scale insects are sap-sucking plant parasites that play an important role in the ecosystem [15]. Honeydew, the waste generated by scale insects feeding exclusively on the phloem sap of host plants, is an important food source for birds, mammals and especially other insects [15]. However, they are common pests of perennial plants in managed systems and may cause chlorosis and leaf fall [16]. Moreover, some armored scale species can prey on more than 100 plant families, including fruit and nut crops, cotton, cereal crops, and forest and ornamental plants [17–20]. They belong to the superfamily Coccoidea and comprise more than 8000 described species from approximately 50 families [15]. Armored scale insects have a global distribution and are the most species-rich taxa in the Coccoidea, comprising more than 2600 species and approximately 400 genera in the family Diaspididae [15]. More specifically, there are 1108 species of scale insects in China, including 452 species of armored scale insects from 82 genera [15]. The Diaspididae possibly comprises the most invasive insect species, as their small size makes them cryptic and difficult to detect and identify [21]. At present, microscopic observations of adult female specimens are the most popular method for identifying armored scale species [22]; however, morphological identification requires an expert, and it cannot distinguish between two closely related species [23]. Additionally, it is difficult to identify species based on the morphological characteristics of specimens from different developmental stages, such as crawlers, second and third instar nymphs, or eggs [24].

DNA barcoding has been used with many taxa in the Coccoidea for primer designing [25], species identification [26–31], genetic diversity estimation [32], and quarantine inspection [33]. However, a search in the Barcode of Life Data System (BOLD) database in July 2023 using the term “Diaspididae” produced 2412 published records, whereas the term “Diaspididae China” produced only 8 records (~0.3%), suggesting a lack of data on the biodiversity of armored scale insects in China.

Fragments of the cytochrome c oxidase subunit I (*COI*) [25, 34] and 28S [35, 36] genes are primarily employed in the identification of armored scale insects. However, some studies suggest that *28S* rDNA lacks sufficient variation to delimitate some species [26, 37]. Therefore, *28S* is considered a complementary marker to *COI* in scale insects [31]. This study aimed to develop a comprehensive DNA barcode library for the armored scale insect species of China as well as assess the accuracy of *COI* barcodes in armored scale insects.

## Materials and methods

### Specimen collection and identification

**Fig 1:**
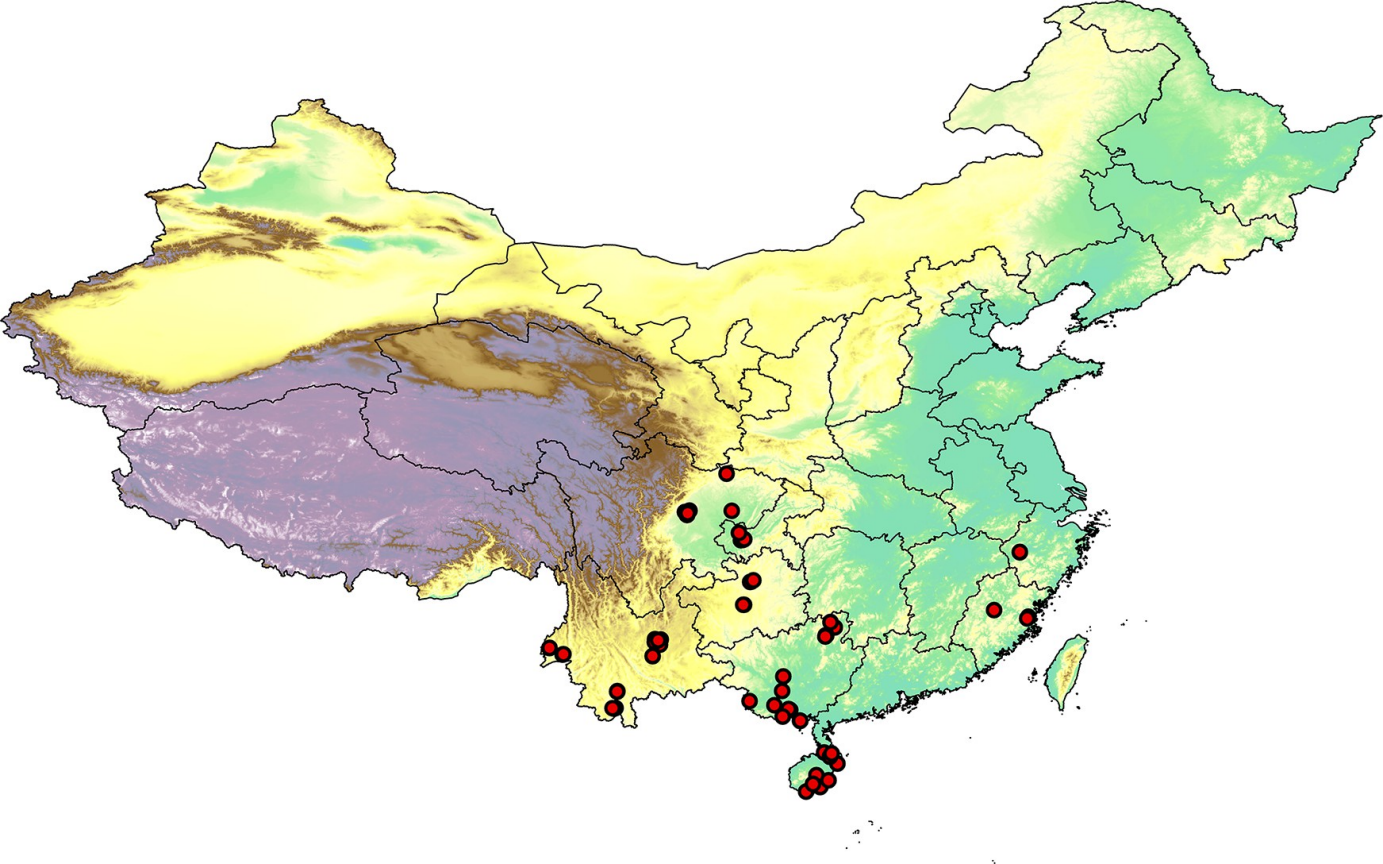
Distribution map of specimens of armored scale insects collected from China during 2021–2022. The basemap was created using the Natural Earth Dataset (http://www.naturalearthdata.com).

A total of 239 armored scale specimens, identified as 50 morphological species, representing two subfamilies and 21 genera were collected from 60 host plant species in seven provinces of southern China (Yunnan, Guangxi, Fujian, Zhejiang, Hainan, Sichuan, and Guizhou) during 2021–2022 (Fig 1). The specimens and their host plant tissues were stored at −20°C for further analysis. Detailed information on each specimen, including location, host plant, and GenBank accession numbers in NCBI (https://www.ncbi.nlm.nih.gov/), is provided in S1 Table. A combined molecular/morphological preparation protocol was performed on each specimen to obtain genomic DNA from the specimens and permanent slides of its cuticle [38]. Morphological identification was conducted independently by Jiufeng Wei and Minmin Niu, according to the morphological studies of the Diaspididae of Williams and Watson [39], Tang [20, 40, 41], Chou [42–44]. Specimen vouchers were deposited in Insect Specimen Museum, College of Plant Protection, Shanxi Agricultural University.

### DNA extraction, PCR, and sequencing

Total genomic DNA of the specimens were extracted using the Ezup column-based animal genomic DNA extraction kit (Shanghai Bioengineering Co., Ltd, Shanghai, China). Thereafter, the DNA samples were used as templates for a PCR amplifying an approximately 650 base pair (bp) fragment of the *COI* barcode region using the universal primers PcoF1 (5’-CCTTCAACTAATCATAAAAATATYAG-3’) [25] and LepR1 (5’-TAAACTTCTGGATGTCCAAAAAATCA-3’) [26]. The 30 μL reaction contained 9.5 μL ddH_2_O, 13.5 μL 2x Taq Master mix (1 mL), 1.5 μL of each primer (10 μM), and 4 μL of template DNA. PCR reaction conditions were as follows: initial denaturation at 95°C for 3 min; followed by 5 cycles of 95°C for 1 min, 48°C for 2 min, and 72°C for 1 min and 35 cycles of 95°C for 1 min, 51°C for 2 min, and 72°C for 1 min; and a final extension at 72°C for 8 min. The PCR products were visualized using 1% agarose gel electrophoresis and sequenced using the forward primer by Qingke Biotechnology Co., Ltd (Shaanxi, China).

### Sequence analysis

The sequencing results were viewed using Chromas v1.62 [45] and sorted using the TBtools software [46]. Subsequently, all sequences were aligned and trimmed using MEGA v11.0 [47] to obtain a matrix of 586-bp long sequences for further molecular analyses.

### Genetic distance and phylogenetic analysis

The intraspecific and interspecific genetic distances were calculated using the Kimura 2 parameter (K2P) [48] and prior intraspecific divergence (P)-distance models and a neighbor-joining (NJ) tree was constructed with the K2P model and 10000 bootstrap replicates in MEGA v11.0. The maximum likelihood (ML) phylogenetic tree was constructed using PhyloSuite v1.2.3 [49] using the following settings: ML + standard bootstrap, 1000 bootstrap replicates, and the TIM + F + I + G4 model obtained by the ModelFinder software [50] under the BIC standard. A maximum parsimony (MP) tree was also constructed with 1000 bootstrap replicates using MEGA v11.0 and the Bayesian inference (BI) phylogenetic tree was constructed with PhyloSuite v1.2.3 using the following settings: 20000000 generations and the GTR+F+I+G4 model obtained by ModelFinder software under BIC standard. Two sequences of *Paracoccus marginatus* (Pseudococcidae, Hemiptera) were used as outgroups (GenBank accession numbers: OR544511 and OR544512). The layout of the NJ tree was edited using the Interactive Tree of Life v6 (https://itol.embl.de/) [51].

### Species delimitation

Several methods have been proposed for species identification based on molecular data [52–56]. This study used the Automated Barcode Gap Discovery (ABGD) (https://bioinfo.mnhn.fr/abi/public/abgd/abgdweb.html) [52], Bayesian Poisson Tree Process (bPTP) [53], Assemble Species by Automatic Partitioning (ASAP) (https://bioinfo.mnhn.fr/abi/public/asap/asapweb.html) [55] and Taxon DNA analysis [56] methods to assess species boundaries and delimit possible species. The ABGD method is a clustering approach based on genetic distances, and it can be performed using different models (JC69, K2P, and P-distance). In the ABGD approach, the relative gap width was set to 1.0 and the P-distance was set to 0.001–0.1. The bPTP method is based on interspecific and intraspecific substitutions, and it assumes that the number of substitutions within a species is lower than the number of substitutions between species. The distinction between species is then achieved by calculating these two values [57]. The ML tree was analyzed on the PTP website (https://species.h-its.org/ptp/) with the following settings: Rooted, Remove outgroups, and default settings for the other parameters. The ASAP analysis is a hierarchical clustering algorithm based on an evolutionary theory that avoids the computational burden of phylogenetic reconstruction by using only pairwise genetic distances [55]. In the ASAP approach, the default settings were selected and data analyzed by three models, Jukes-Cantor (JC69), Kimura (K80) ts/tv 2.0, and Simple Distance (p-distances). For the Taxon DNA Analysis method, parameters were set to Best match, Best close match, All species barcodes and Cluster methods to analyze the *COI* sequences.

## Results

### Genetic distance and phylogenetic analysis

Analysis of the sequencing data revealed 222 conserved sites, 364 variable sites, 334 parsimony information sites, and 30 singleton sites accounting for 37.88%, 62.12%, 56.99%, and 5.12% of the total *COI* gene sequence (586-bp), respectively. The average thymine (T), cytosine (C), adenine (A), and guanine (G) contents of the *COI* gene sequences were 41.32%, 11.44%, 40.62%, and 6.62%, respectively. The average A + T and G + C contents of the *COI* gene sequences were 81.94% and 18.06%, respectively, indicating a strong A + T bias.

**Fig 2:**
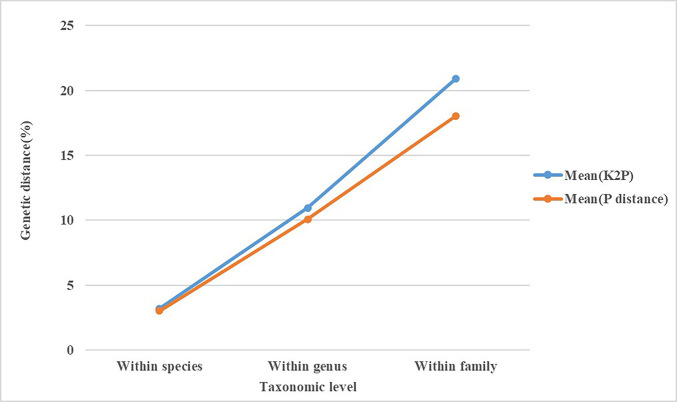
Line chart of the mean genetic distances of the cytochrome oxidase subunit I (*COI*) gene sequences of armored scale insects from China at different taxonomic levels based on the Kimura 2 parameter and prior intraspecific divergence (P) distance models.

**Fig 3:**
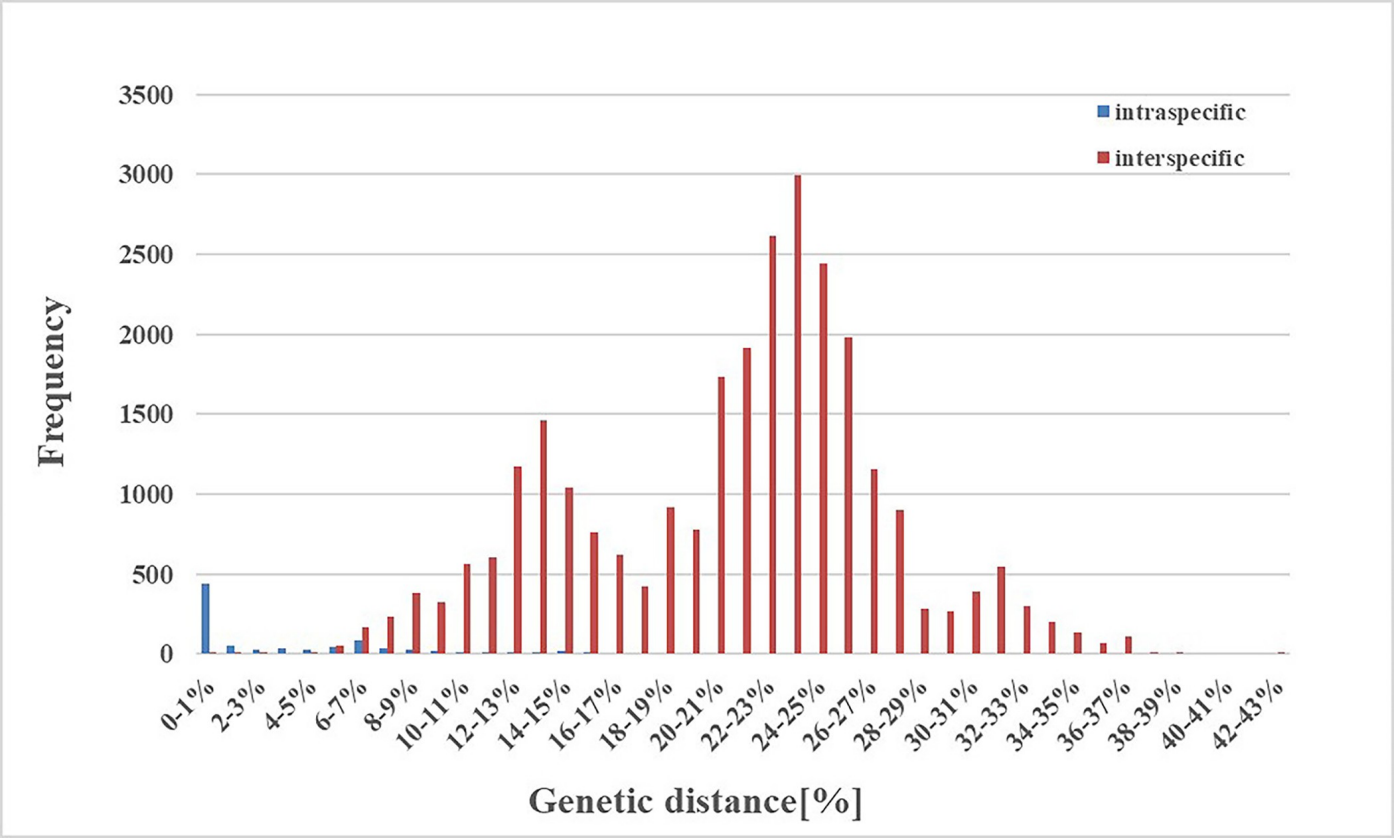
Frequency histogram of intraspecific and interspecific genetic distances of armored scale species from China, based on the cytochrome c oxidase subunit I (*COI*) sequences.

**Fig 4:**
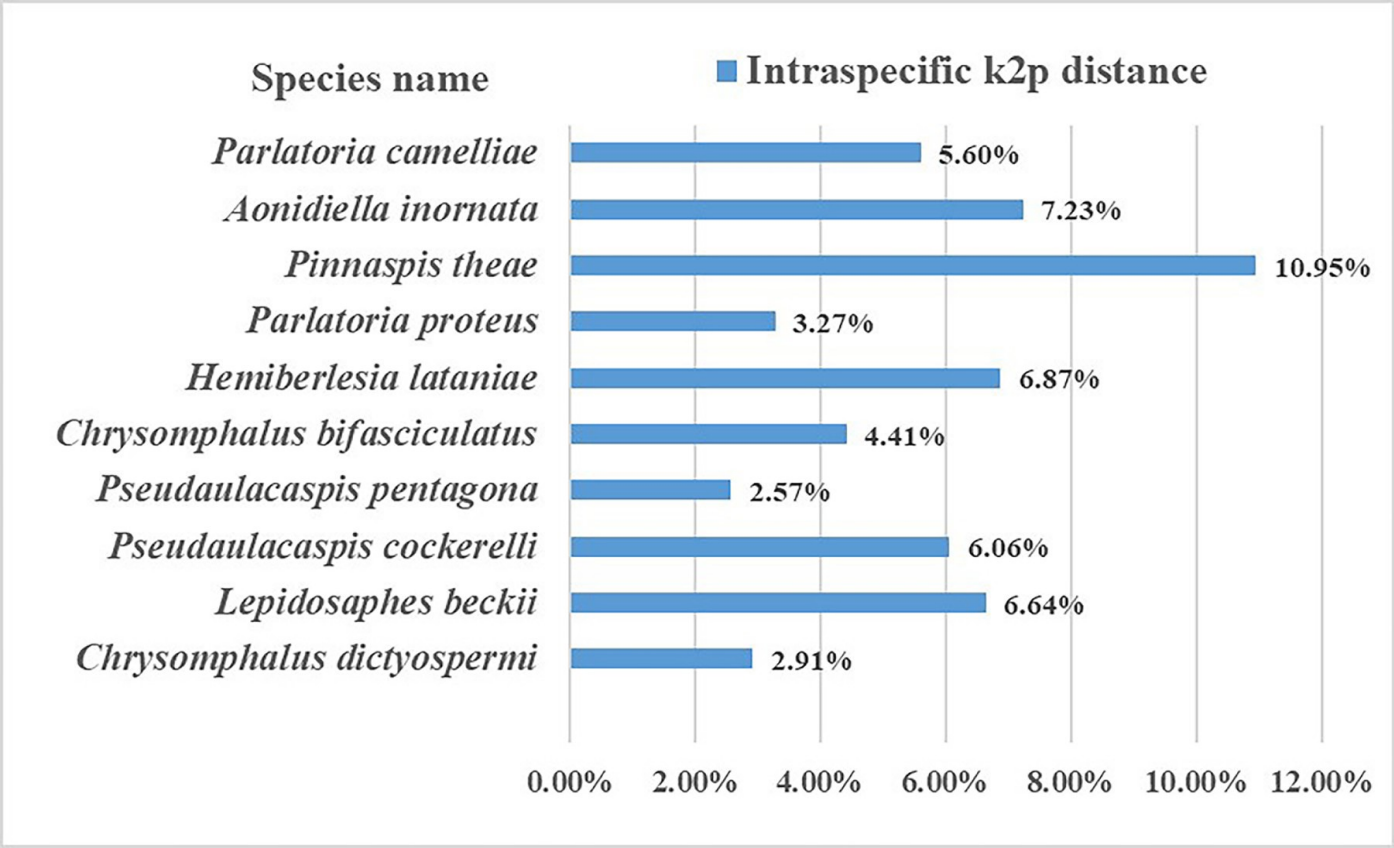
Ten armored scale species from China with > 2% intraspecific Kimura 2 parameter distances based on the cytochrome oxidase subunit I (*COI*) gene sequence.

As expected, the mean genetic distances increased hierarchically with taxonomic categories based on the K2P and P-distance models. As seen in Fig 2, the genetic distances calculated by the K2P model were only slightly higher than those calculated by the P-distance model, with intraspecific, interspecific, and intergeneric distances of 3.20% and 3.02%; 10.94% and 10.07%; 20.91% and 18.03%, respectively. Since the results of the two models were very similar, the K2P distance model was used for further analysis. The intraspecific K2P distances ranged from 0 to 15.34%, and approximately 68.43% of the intraspecific distances were < 5%, while 99.88% of the interspecific distances were > 5%. This suggests an obvious barcoding gap in the data (Fig 3). Also, 10 species had K2P distances greater than 2% (S2 Table and Fig 4).

Based on the data in Table 1 and Fig 3, the intergeneric distance threshold of ≤ 5% was appropriate for the differentiation of Diaspididae in China, where intergeneric distances > 5% suggest the presence of cryptic species.

**Table 1:** Kimura 2 parameter genetic distances (%) based on the cytochrome oxidase subunit I gene (*COI*) of the armored scale insects from China at different taxonomic levels.

Taxonomic level	Cytochrome oxidase subunit I		
	Minimum (%)	Mean (%)	Maximum (%)
Intraspecific distance	0.00	3.20	15.34
Interspecific distance	5.39	10.94	19.90
Intergeneric distance	5.39	20.91	36.89

**Fig 5:**
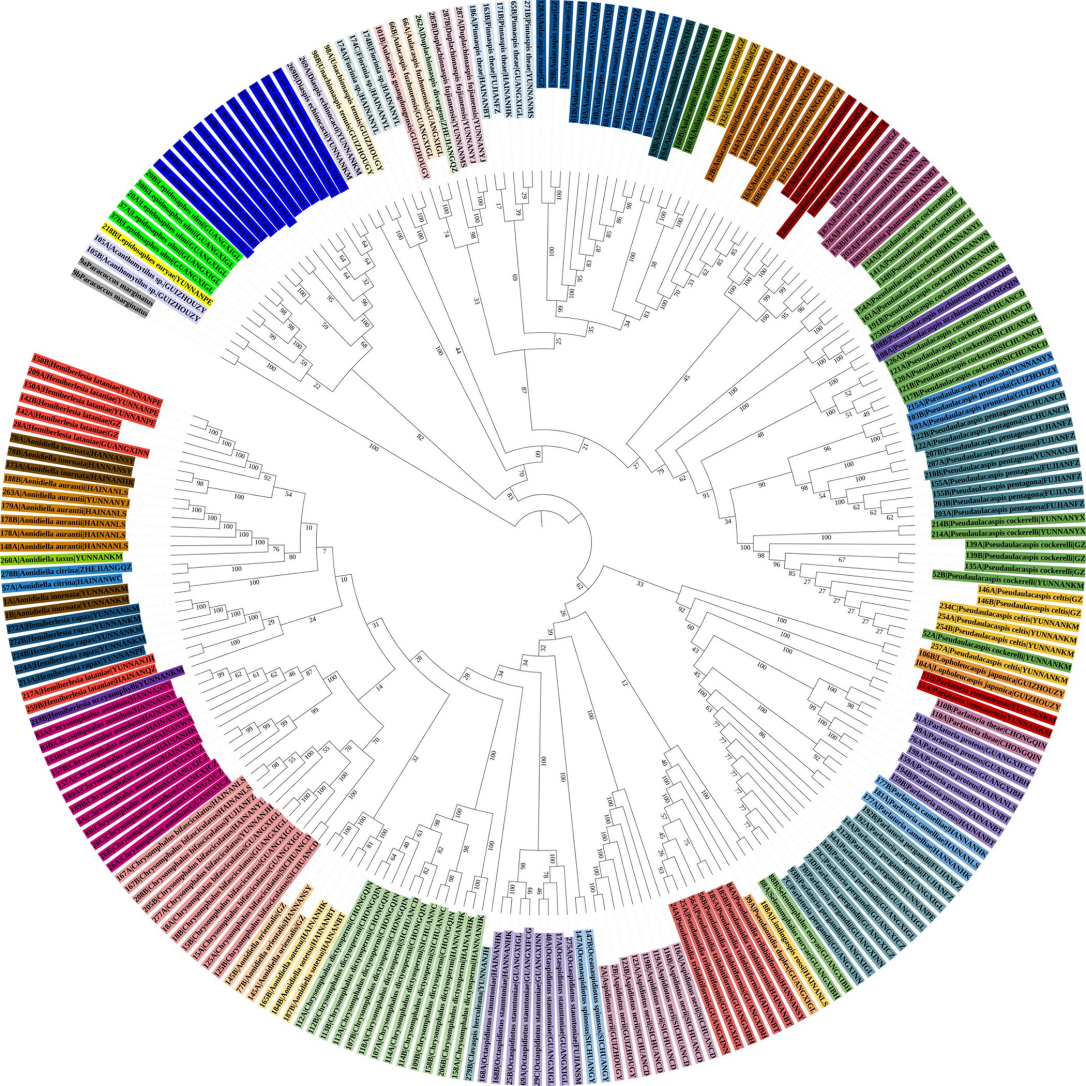
Radial chronogram of the delimited armored scale insect species from China. The backbone represents the neighbor-joining (NJ) tree based on the cytochrome c oxidase subunit I (*COI*) gene sequences, and the colored circles represent different morphospecies.

**Fig 6:**
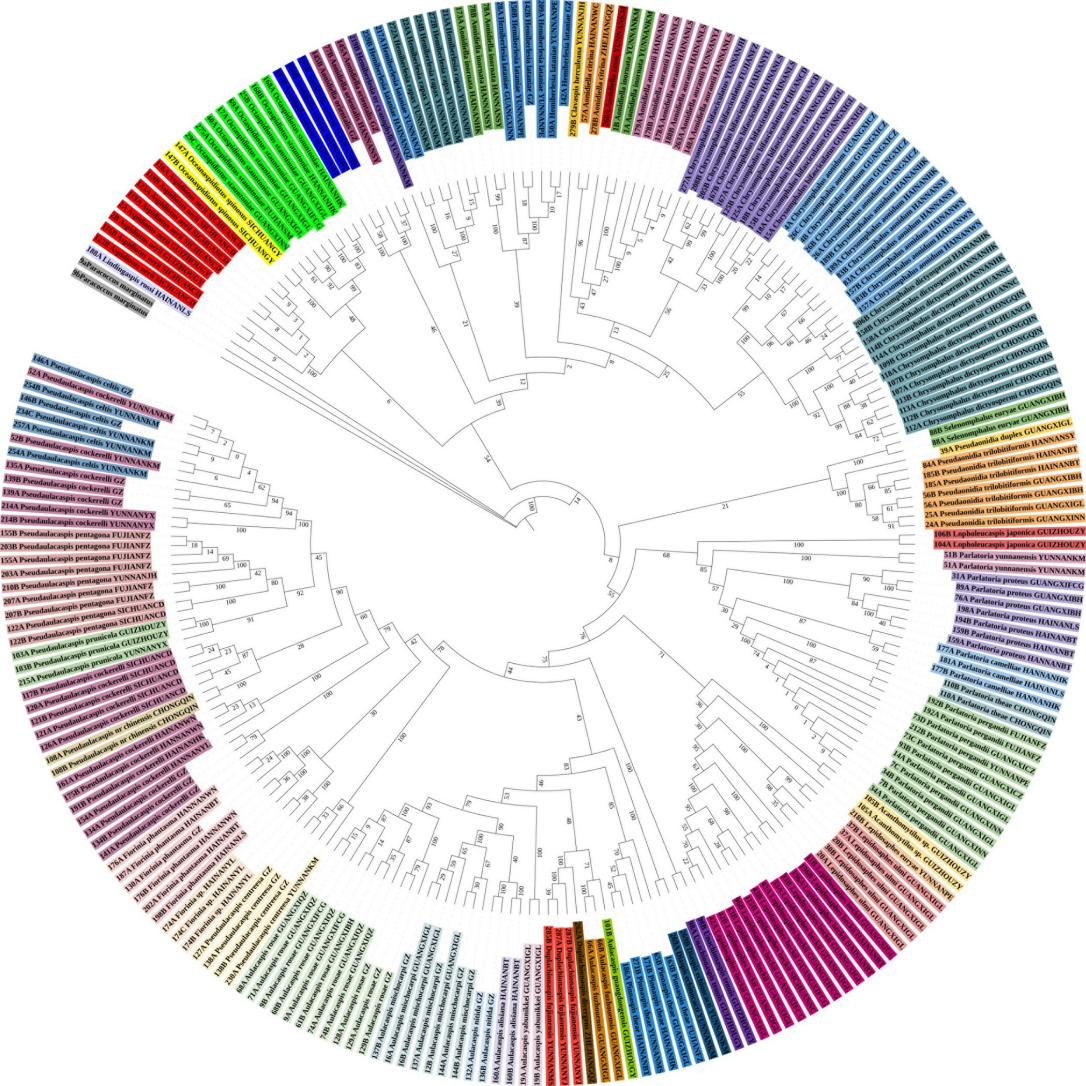
Radial chronogram of the delimited armored scale insect species from China. The backbone tree represents the maximum likelihood (ML) tree based on the cytochrome c oxidase subunit I (*COI*) gene sequences, and the colored circles represent different morphospecies.

The NJ tree constructed from the 239 *COI* sequences can be seen in Fig 5. A total of 46 species formed monophyletic branches with a high bootstrap support at the species level, which was consistent with traditional taxonomic results. The ML tree constructed from the 239 *COI* sequences can be seen in Fig 6. A total of 46 species formed monophyletic branches at the species level with high bootstrap support, which was consistent with traditional taxonomic results.

**Fig 7:**
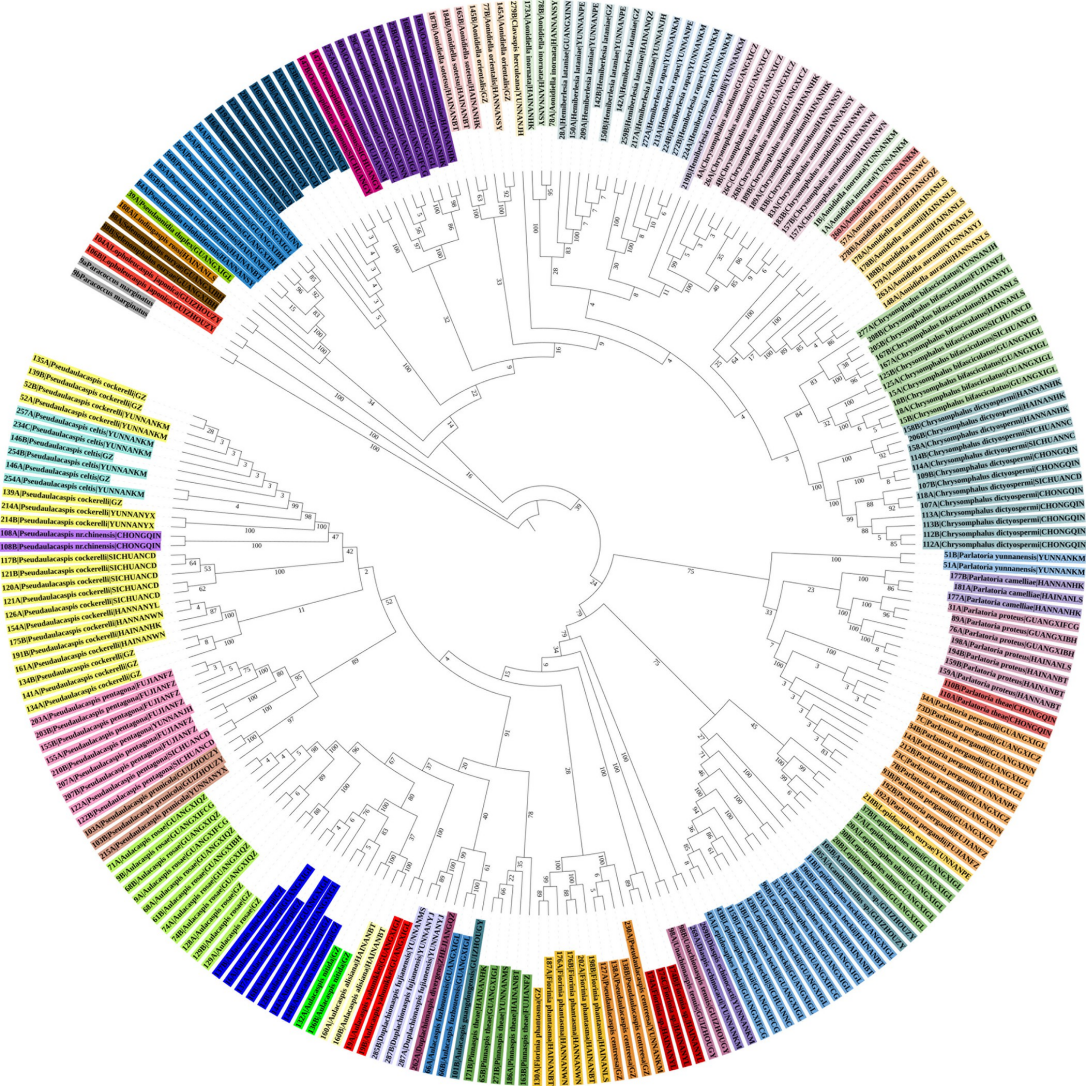
Radial chronogram of the delimited armored scale insect species. The inner backbone tree represents the maximum parsimony (MP) tree based on the cytochrome c oxidase subunit I (*COI*) gene sequence, and the colored circles represent different morphospecies.

**Fig 8:**
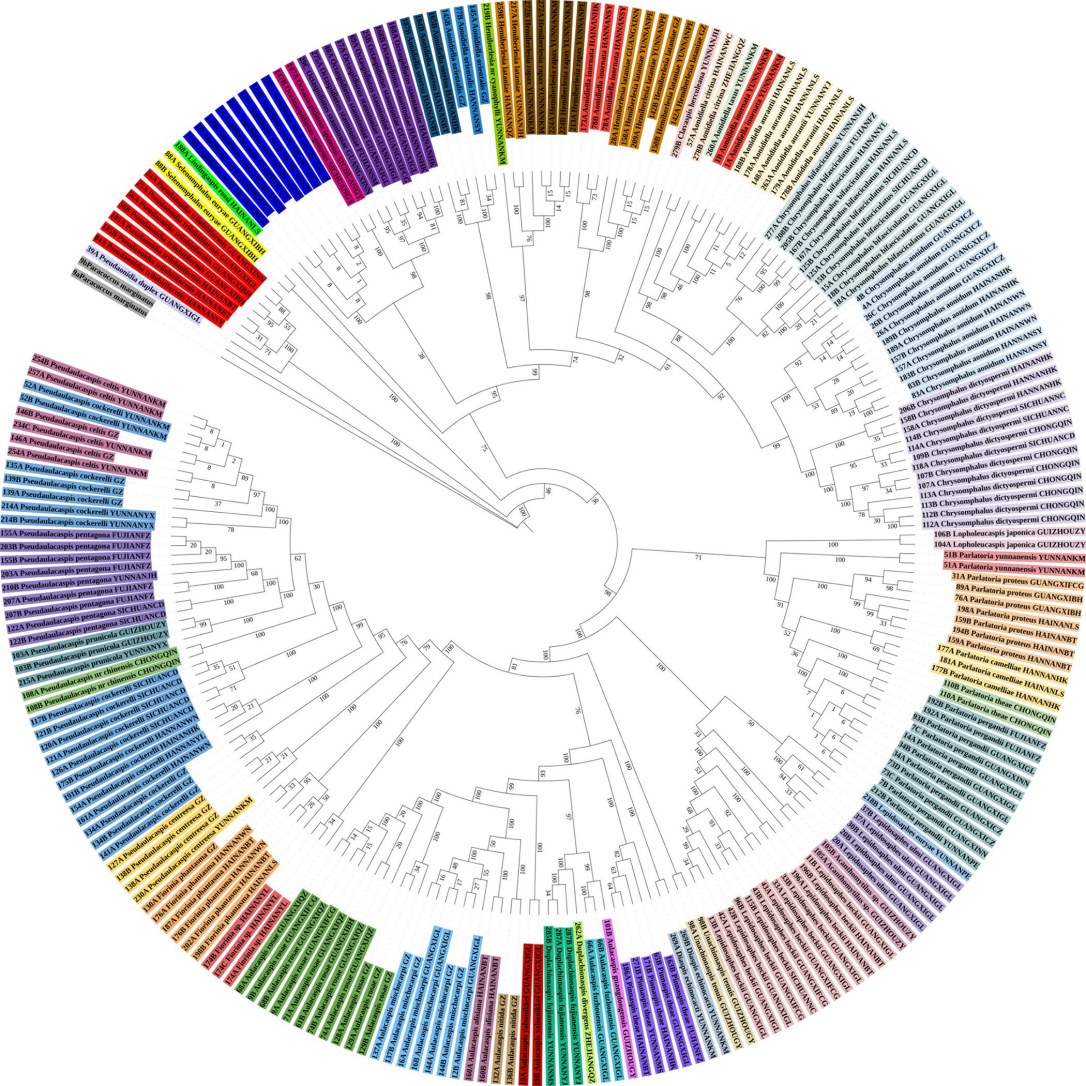
Radial chronogram of the delimited armored scale insect species from China. The inner backbone tree represents the Bayesian inference (BI) tree based on the cytochrome c oxidase subunit I (*COI*) gene sequences, and the colored circles represent different morphospecies.

The MP tree constructed from the 239 *COI* sequences is shown in Fig 7. A total of 46 species formed monophyletic branches at the species level with high bootstrap support, which was consistent with traditional taxonomic results. The BI tree constructed from the 239 *COI* sequences showed a total of 46 species on monophyletic branches at the species level with high bootstrap support, which was consistent with traditional taxonomic results (Fig 8).

### Species delimitation

The results of the ABGD analysis performed using the JC69, K2P, and P-distance models were compared and data revealed that the number of groups based on the P-distance model ranged from 66 to 75, and the initial partition engendered 66 groups (P = 0.0028–0.0359) (Table 2) (S3 Table). The ABGD analysis categorized 13 sequences of *Chrysomphalus dictyospermi* into two groups, 12 sequences of *Lepidosaphes beckii* into three groups, 19 sequences of *Pseudaulacaspis cockerelli* into four groups, 11 sequences of *Chrysomphalus bifasciculatus* into four groups, eight sequences of *Hemiberlesia lataniae* into three groups, five sequences of *Pinnaspis theae* into five groups, five sequences of *Aonidiella inornata* into two groups, three sequences of *Parlatoria camelliae* into two groups, and six sequences of *Pseudaulacaspis celtis* along with seven sequences of *P*. *cockerelli* into one group. These results indicate a species classification success rate (classification of the same species in the same group) of 65.69% for the ABGD analysis.

**Table 2:** ABGD analysis of *COI* sequences of armored scale insect species from China, based on three evolutionary models.

Model	X	Partition	Prior intraspecific divergence (P)							
			0.001	0.0017	0.0028	0.0046	0.0077	0.0129	0.0215	0.0359
JC69	1	Initial	66	66	66	66	66	66	66	66
		Recursive	92	92	83	77	75	73	71	66
K2P	1	Initial	66	66	66	66	66	66	66	66
		Recursive	93	93	83	77	75	73	71	66
P	1	Initial	66	66	66	66	66	66	66	66
		Recursive	75	75	75	72	72	69	69	66

Table 3 shows the results of the ASAP analysis under the three models. The lower the ASAP score, the more reliable the partitioning results. The partitioning of the K2P model was closest to the morphological results (S4 Table). The ASAP analysis categorized 13 sequences of *C*. *dictyospermi* into two groups, 12 sequences of *L*. *beckii* into three groups, 19 sequences of *P*. *cockerell* into four groups, 11 sequences of *C*. *bifasciculatus* into four groups, 11 sequences of *Aulacaspis rosae* into two groups, eight sequences of *H*. *lataniae* into three groups, seven sequences of *Parlatoria proteus* into three groups, five sequences of *P*. *theae* into five groups, and six sequences of *P*. *celtis* along with seven sequences of *P*. *cockerelli* into one group. These results indicate a species classification success rate of 61.51% for the ASAP analysis.

**Table 3:** ASAP analysis of *COI* sequences of armored scale insect species from China, based on three evolutionary models.

Model	ASAP scores -10 best partitions									
JC69	5.50	7.00	7.50	7.50	8.50	9.00	11.00	11.50	11.50	13.50
	-70	-70	-73	-74	-71	-79	-69	-68	-70	-70
K2P	7.50	9.00	11.00	11.50	11.50	12.00	13.00	14.00	14.50	17.00
	-68	-70	-73	-71	-74	-78	-61	-83	-66	-72
P	6.00	7.00	7.00-	8.00	8.50	9.00	11.00-	11.00	11.50	13.50
	-70	-70	74	-73	-71	-79	67	-68	-69	-54

In the Taxon DNA analysis, the threshold of 239 *COI* sequences was calculated to be 6.82% by the Pairwise Summary method. With the Best match method, the number of accurately identified sequences was 222, with a success rate of 92.88%; the number of ambiguous sequences was 10, accounting for 4.18% of all the sequences; the number of incorrect identifications was seven, accounting for 2.92%. For the Best close match method, the number of accurately identified sequences was 215, with a success rate of 89.95%; the number of ambiguous sequences was eight, accounting for 3.34% of all sequences; the number of incorrect identifications was three, accounting for 1.25%; the number of sequences without any match closer than 6.82% was 13, accounting for 5.43%. With the All Species Barcodes method, the number of accurately identified sequences was six, the success rate was 2.51%; the number of ambiguous sequences was 218, accounting for 91.21%; the number of incorrect identifications was two, accounting for 0.83%, and the number of sequences with no match closer than 6.82% was 13, accounting for 5.43%.

The Cluster method divided 239 sequences into 70 groups with a default threshold of 3% (S5 Table). The Cluster analysis categorized 13 sequences of *C*. *dictyospermi* into two groups, 12 sequences of *L*. *beckii* into three groups, 19 sequences of *P*. *cockerell* into four groups, 11 sequences of *C*. *bifasciculatus* into four groups, 11 sequences of *Aulacaspis rosae* into two groups, eight sequences of *H*. *lataniae* into three groups, seven sequences of *Parlatoria proteus* into three groups, five sequences of *P*. *theae* into five groups, five sequences of *A*. *inornata* into two groups, three sequences of *P*. *camelliae* into two groups, and six sequences of *P*. *celtis* along with seven sequences of *P*. *cockerelli* into one group. These results indicate a species classification success rate of 58.16% for the Cluster analysis.

A total of 74 putative species were delimited using the bPTP analysis (S1 Fig). The bPTP analysis categorized five sequences of *P*. *theae* into five groups, 12 sequences of *L*. *beckii* into three groups, five sequences of *A*. *inornata* into two groups, eight sequences of *H*. *lataniae* into three groups, three sequences of *P*. *camelliae* into three groups, 19 sequences of *P*. *cockerell* into five groups, 13 sequences of *C*. *dictyospermi* into two groups, 11 sequences of *C*. *bifasciculatus* into four groups, nine sequences of *Pseudaulacaspis pentagona* into three groups, 11 sequences of *Aulacaspis rosae* into two groups, seven sequences of *Parlatoria proteus* into three groups, three sequences of *Pseudaulacaspis prunicola* into two groups, and six sequences of *P*. *celtis* along with five sequences of *P*. *cockerelli* into one group. These results indicate a species classification success rate of 53.14% for the bPTP analysis.

## Discussion

Paul Hebert first introduced the concept of DNA barcoding in 2003 and suggested that the *COI* mitochondrial gene can be used as a universal barcode to identify all animals [1, 2]. Thereafter, *COI*-based DNA barcoding has been applied to delineate species in a wide range of animal taxa [58–63], by primarily utilizing the apparent gaps in genetic distances between the *COI* sequences of different species. After analyzing 13320 organisms, Herbert et al. [1, 2] proposed an intraspecific genetic distance of ≤ 2%, which is still widely accepted. However, studies have found that the thresholds of genetic distance were not completely uniform across species [64]. The current identification threshold for the BOLD database is 3% [65] but specific species boundary thresholds have been identified for many insects. For instance, Ball & Armstrong [66] obtained a 12.8% interspecific divergence for the New Zealand sooty beech scale insect, while Park et al. [26] found an average of 10.7% interspecific divergence and 0.97% intraspecific divergence for the Pseudococcidae and Diaspididae using *COI*-based DNA barcoding.

This study used five methods to determine whether *COI*-based DNA barcoding is effective in delimiting armored scale insect species from China. The K2P model provided a maximum intraspecific genetic distance of 15.34%, which significantly exceeds the 2% threshold proposed by Hebert and the 3% threshold of the BOLD database. Results revealed a significant overlap between the intraspecific and interspecific genetic distances, which may be because of insufficient data or inaccurate morphological identification of samples. However, these results are nonetheless informative for determining Diaspididae thresholds. The ABGD analysis showed a 65.69% species delineation success rate, does not require any input on genetic distance, and the results can be seen in its delineation of the threshold range of the species, to provide a reference for the selection of the correct threshold. The ASAP analysis showed a 61.51% species delineation success rate and is simple and easy to use, with clear results. For the Taxon DNA Analysis, the accurate identification rates of the Best match, Best close match and All species barcodes methods were 92.88%, 89.95% and 2.51%, respectively. The Cluster analysis showed a 58.16% species delineation success rate and is a commonly used method for analyzing the success rate of DNA barcoding [56]. The bPTP analysis showed a 53.14% success rate of species delineation and requires a prior input of a phylogenetic tree for species delineation, which is complicated and time-consuming.

In these analyses, most of the species could be clustered into groups. Therefore, a combination of genetic divergence analysis along with the NJ, ABGD, ASAP, Taxon DNA and bPTP analyses may help in the accurate identification of armored scale insect species. Based on the results of this study, an intergeneric distance of ≤ 5% was considered an appropriate threshold for the identification of the Diaspididae in China using the *COI* gene region. The NJ tree, ML tree, MP tree and BI tree all showed a total of 46 species, forming monophyletic branches at the species level.

In this study, fresh and full-bodied female armored scale insect specimens were collected and molecular and morphological analyses performed. The morphological integrity of the insects was maintained after genomic DNA extraction, which played an important role in post-experimental morphological review and species preservation. Although the obtained DNA concentration of the specimens was not high, it was sufficient for PCR amplification. This suggests that DNA barcoding can even be performed using small quantities of DNA from morphologically indistinguishable or mutilated specimens. This adds advantage to the delineation of cryptic species, where cryptic species diversity is being revealed by the development and application of DNA classification methods. This study found large genetic distances between the specimens that were identified as the same species, suggesting the presence of cryptic species.

Currently, it is widely believed that classification should reflect phylogeny [38]. The phylogenetic results obtained here are consistent with Normark et al., [38], with species mainly divided into the Diaspidinae and Aspidiotinae with the same level of strong statistical support observed for the species in both molecular phylogenetic trees. However, *P*. *centreesa* comprised a clade separate from the genus *Pseudaulacaspis*, and from a morphological perspective, the biggest difference between *P*. *centreesa* and other species of the genus *Pseudaulacaspis* is that its glandular spines are much longer. So, this species may not belong to the *Pseudaulacaspis* genus.

The main difference in morphology between *P*. *celtis* and *P*. *cockerelli* is that *P*. *celtis* has gland tubercles on the mesothorax and fewer dorsal macroducts (there are four pairs in *P*. *celtis* and five *pairs* in *P*. *cockerelli* in the 2nd stage female) [20]. However, *P*. *celtis* and some *P*. *cockerelli* group together in the phylogenetic analyses. Possible reasons for this include the fact that genes are extracted from female adults which cannot be compared with immature or male morphologies; and the presence or absence of dorsal ducts and the number of dorsal ducts are not the main distinguishing features in the submedian area of the second abdominal segment of *P*. *celtis*. Phylogenies also revealed that *Aspidiotinae*, *Chrysomphalus*, *Aonidiella* and *Hemiberlesia* are non-monophyletic and overlaps, forming a single clade. This suggests additional molecular and morphological work is needed for these genera.

The purpose of this study was to supplement the DNA barcoding library of armored scale insects in China, which can greatly improve the identification of scale insects, including immature and male scale insects. The results showed that *COI*-based DNA barcoding is a rapid and accurate technique for the identification of armored scale insect species. However, at present, *COI*-based DNA barcoding cannot be used independent of morphological analysis, and the two techniques should be combined for the accurate and efficient identification of armored scale insect species. Additionally, multi-molecular markers can also be used for species identification to improve accuracy.

## Conclusion

This work increased the number of *COI* sequences available for common armored scale insects from China by adding 239 *COI* sequences from 50 morphological species representing 21 genera and two subfamilies. Analyses employed the Automated Barcode Gap Discovery, Assemble Species by Automatic Partitioning, Taxon DNA analysis and Bayesian Poisson Tree Process methods, which yielded largely consistent results, indicating a robust and credible species delimitation. Based on these results, an intergeneric threshold of ≤ 5% is recommended for the identification of the Diaspididae in China. However, individual morphospecies may exist with cryptic species and more work is needed to elucidate these issues. Therefore, this study provides novel insights into the identification of armored scale insects in China and provides a DNA barcode library for future research and application.

## Supporting information

S1 Table Sample list and collection information of the 239 specimens, including accession numbers in NCBI (https://www.ncbi.nlm.nih.gov/), specimen ID, host plant, and collect information. OR544511 and OR544512 are outgroups downloaded from GenBank.
(XLSX)

S2 Table The intraspecific and interspecific genetic distances of congeneric species of armored scale insects. The genetic distances were calculated based on the K2P model. The intraspecific genetic distances were calculated when a species had at least two individuals. The interspecific genetic distances were calculated when a genus had at least two species.
(XLSX)

S3 Table Results of the automatic barcode gap discovery (ABGD) analyses. (XLSX)

S4 Table Results of the assemble Species by Automatic Partitioning (ASAP) analyses. (XLSX)

S5 Table Results of the cluster method. (XLSX)

S1 Fig Results of the bayesian Poisson Tree Process (bPTP) analyses. (JPG)
